# “Lessons from the pandemic: How can we enhance surgical training in undergraduate education?"

**DOI:** 10.1016/j.amsu.2022.103900

**Published:** 2022-05-31

**Authors:** Jonathan Roy Honey, Hannah Rebecca Mudge

**Affiliations:** a The University of Cambridge School of Clinical Medicine, Addenbrooke's Hospital, Hills Rd, Cambridge, CB2 0SP, UK; b The University of Southampton School of Medicine, 12 University Rd, Southampton, SO17 1BJ, UK

## 1 “Lessons from the pandemic: how can we enhance surgical training in undergraduate education?"

The pandemic has had significant repercussions for the way undergraduate surgical education has been taught over the last two years. To prevent a workforce or skills shortage in new graduates, it is essential to learn from the limitations covid-19 imposed on opportunities for developing the outcomes required by the GMC. By innovating in light of the recent challenges, we can enhance surgical training both now and for the future.

## 2 Assessing the damage: highlighting the lessons from the pandemic

The impact of Covid-19 on medical school education has been explored in numerous papers, though fewer focused specifically on surgical training or possible enhancements. [[1], [2], [3], [4],[4], [4], [5], [6], [7]] [[8], [9], [10], [11], [12]] The leading negative effects on undergraduate surgical training identified can be divided into three. Firstly, there has been reduced time in theatres, clinics and on wards, leading to reduced operative (surgical scrub, suturing, hand ties, laparoscope assistance) and non-operative (ward craft, history taking, examinations, communication, leadership, teamwork) knowledge and skills. Secondly, there has been reduced opportunity for portfolio building, including the cancellation of surgical electives, conferences, intercalations, and research projects – which will inequitably disadvantage certain groups. Finally, surveys have indicated reduced confidence in surgical ability as well as reduced mental health and enthusiasm for medicine in undergraduates, which may lead to future staff shortages. This is compounded by reduced “apprenticeship”-style mentoring opportunities, such as the life advice from surgeon colleagues that helps shape which specialty a student decides. Let us consider the findings in greater detail, suggesting methods of enhancement to training to overcoming these.

## 3 Reduced clinical exposure leading to inexperience

Surgical placements are an essential component of a medical student's education. [13,14] Surgical inexperience has increased through the pandemic as access to clinical environments became severely limited and substituted for online teaching of varying quantity and quality. [15] [1,7,12,16,17] A logbook review comparing 2019 to 2020 found a “50% reduction in operations with trainees as the primary operating surgeon”. [18] It is likely the number of operations with medical students observing or assisting had an even more drastic reduction. The substituted resources in general were didactic lectures and seminars, rather than simulation training – which is considered to be superior for developing surgical skills. [19,20] Adapting face-to-face learning experiences (theatres, wards, lab work) into a virtual format is one of the greatest challenges facing surgical education. [8]

One innovative way to achieve this would be to create a bank of augmented reality (AR) learning resources that simulate ward work and theatre. Watching the operation (live or recorded) would be the next best thing for a student unable to get into theatre. If recorded through a camera attached to the surgeon's head, an operation could take the student through from the first incision to skin closure. The video could pause at key moments to ask questions, such as “name this structure”, “what are we cutting through?“, and “why must we be careful here?“. A similar concept using live AR on a ward round has been trialled for education at St George's Hospital, London, and was very positively received. [21] The RCS of Edinburgh have also encouraged remote operation observation. [22] A less-data intensive but less realistic method has already been deployed by the Leiden university centre of innovation with animated surgical cartoon videos. [23] These video-based interactive learning resources mean students could go at their own pace and explore further reading more easily. Of course, this concept necessitates ensuring patient confidentiality, consent, and data protection. It would also not replace the benefit of undergraduates assisting in theatres; nevertheless, it would prove an incredibly effective educational tool enabling students to maximise the value of in-person education when it is possible. This blended style of teaching could be the future of surgical education. [24]

Other ideas for overcoming inexperience include providing beyond-syllabus opportunities for keen final year students to work as an assistant, for instance in a transplant rota sign-up. An extra-curricular surgical skills course could be provided prior to signing up to ensure the necessary skills baseline. Thanks to advances in video technology, this could feasibly be provided virtually – even courses like the RCS Surgical Skills for Students Course. By providing a student with the materials for sutures, undergraduates could be instructed in different stitches and hand ties over a video call group lesson. This could even be integrated into a vignette with an evolving case which asks the student how they would manage the patient beyond the suturing – which could even work as a new exam format. Unfortunately, the challenge comes when a student gets stuck on a technique and is unable to have in-person guidance to rectify. There are also limitations in peer-to-peer learning in the virtual format. This could lead to fewer of the mental health benefits of in-person social learning. [25] Nevertheless, this could be an exciting new method of enhancing surgical undergraduate training.

## 4 Inequalities in access

Secondly, it is worth emphasising the importance of equality of opportunity for undergraduates wishing to pursue surgery. Competition for Core Surgical Training is at its highest in the last 10 years [26,27] Whilst it might be thought that “every undergraduate was in the same boat” through covid with placements cancelled nationally, it is clear that the levels of opportunity and quality of teaching at different universities were vastly different – described by the BMA as a “disparate … mixed bag”. [7] The pandemic also meant those with personal connections to surgeons could more easily boost their portfolio by offering to help with projects than those at home with either poor internet or difficult home situations. Educational opportunity needs to be maximised for all undergraduates and Covid-19 has exacerbated the inequalities of opportunity for pursuing a surgical career (a career which is already behind multiple paywalls).

One effective method to combat this inequality would be to develop a national database of surgical opportunities. This should include skills courses, essay and anatomy competitions, conferences, best elective locations, interesting new surgical breakthroughs, and research advertisements requesting for keen medical students. This would provide equality of access to portfolio building opportunities.

Furthermore, perhaps the single most effective method to enhance surgical training for all students, and not just those with an interest, would be a nationally accessible initial lecture on ‘An Introduction to Surgery’. This should include how theatres run and theatre etiquette so that all students feel confident to participate actively in their surgical placements. There is already a standardised surgical curriculum that mandates specific learning objectives and this lecture could draw from this. [13,14] An introductory webinar to surgery and its trajectory (e.g., details on CST) could inspire more brilliant candidates towards surgery but even more so, improve outcomes for those not interested in surgery [28].

## 5 Student disinterest and burnout

Lost clinical opportunities, particularly in theatres, risks uninspiring surgical rotations. Undergraduates and foundation year doctors are at a unique stage along the development of surgeons and physicians. They are, due to lack of experience, “undifferentiated”, and a good placement can shape whether they decide to pursue one particular specialty over another. For registrars, surgical training has been slowed by the pandemic (c.f. Munro, BMJ); [18]; however, for undergraduates the “career trajectory” may have been redirected entirely for those whose surgical placement was cancelled or severely limited. To avoid this, University surgical societies could play a very important role in offering students access to a mentor scheme – whereby the society pairs a student with a junior surgeon who is happy to impart their surgical (and life) advice. YouTube can play a role too, with doctors on the surgical pathway share their experiences and reasons why they love surgery (c.f. ‘Dr Jude’). [29]

Academic engagement has been shown to be an important protective factor against burnout and dropout intention at medical school [4]; a poor surgical placement has the potential for fewer students to choose surgery down the line or, even more sadly, for students to drop out of medicine entirely having not found their “niche”. [3,30,31] This is in the face of a growing backlog of surgeries and therefore a greater need for surgeons and doctors. As such, any enhancement to surgical education for undergraduates needs to consider preventing disinterest and burnout. National and international connections to surgeons who love their job, fun competitions (“learning to thread a string through a polo mint using laparoscopic tools”) and providing opportunities for students to work as assistants (as above) will all help to inspire and kindle a passion for surgery.

## 6 Conclusion

The adage goes, “if it ain't broke, don't fix it.“. The pandemic has ‘broken’ a system that was previously reliably producing students with an interest in surgery and who are competent in basic surgical skills. If one cannot get into theatre, it is much harder to develop passion or knowhow for surgery. The enhancements above address the three primary “wounds” to surgical education in the last two years and would significantly enhance undergraduate passion, confidence, ability and opportunity.

## 7 Annals of Medicine and Surgery

The following information is required for submission. Please note that failure to respond to these questions/statements will mean your submission will be returned. If you have nothing to declare in any of these categories then this should be stated.

## Ethical approval

No ethics approval was necessary.

## Please state any sources of funding for your research

This short communication was awarded first place in the 'ASiT x Annals of Medicine and Surgery x RCSEng Medical Student Essay Prize 2022'. The prize for first place was £150 and funding for publication. The decision to submit the article for publication, and the funding for publication, was provided by this collaboration between The Association of Surgeons in Training (ASiT), the Annals of Medicine and Surgery and the Royal College of Surgeons of England.

This short communication is the sole work of the authors. The Essay Prize sponsors provided the short communication title; but otherwise they were not involved in preparation of the article in any way.

## Author contribution

**Jonathan R. Honey** – essay ideas and structure, data collection in the form of a literature review, wrote the paper: first (and corresponding) author. **Hannah R. Mudge** – scholarly discussion on ideas and structure as well as critical review and proofread: second author.

## Please state any conflicts of interest

No conflicts of interest.

## Consent

No patient consent was required for this essay.

## Registration of research studies

1. Name of the registry:
2. Unique Identifying number or registration ID:
3. Hyperlink to your specific registration (must be publicly accessible and will be checked):

## Guarantor

Jonathan Honey accepts full responsibility for the work.

## Appendix A Supplementary data

Supplementary Figure One a selection of enhancements placed sequentially along the medical school progression timeline. Supplementary Figure One
