# Supplementary figures and images for: “Lessons from the pandemic: How can we enhance surgical training in undergraduate education?"
Source: Ann Med Surg (Lond). 2022 May 31;79:103900. doi: 10.1016/j.amsu.2022.103900 (PMC9153175; doi:10.1016/j.amsu.2022.103900)

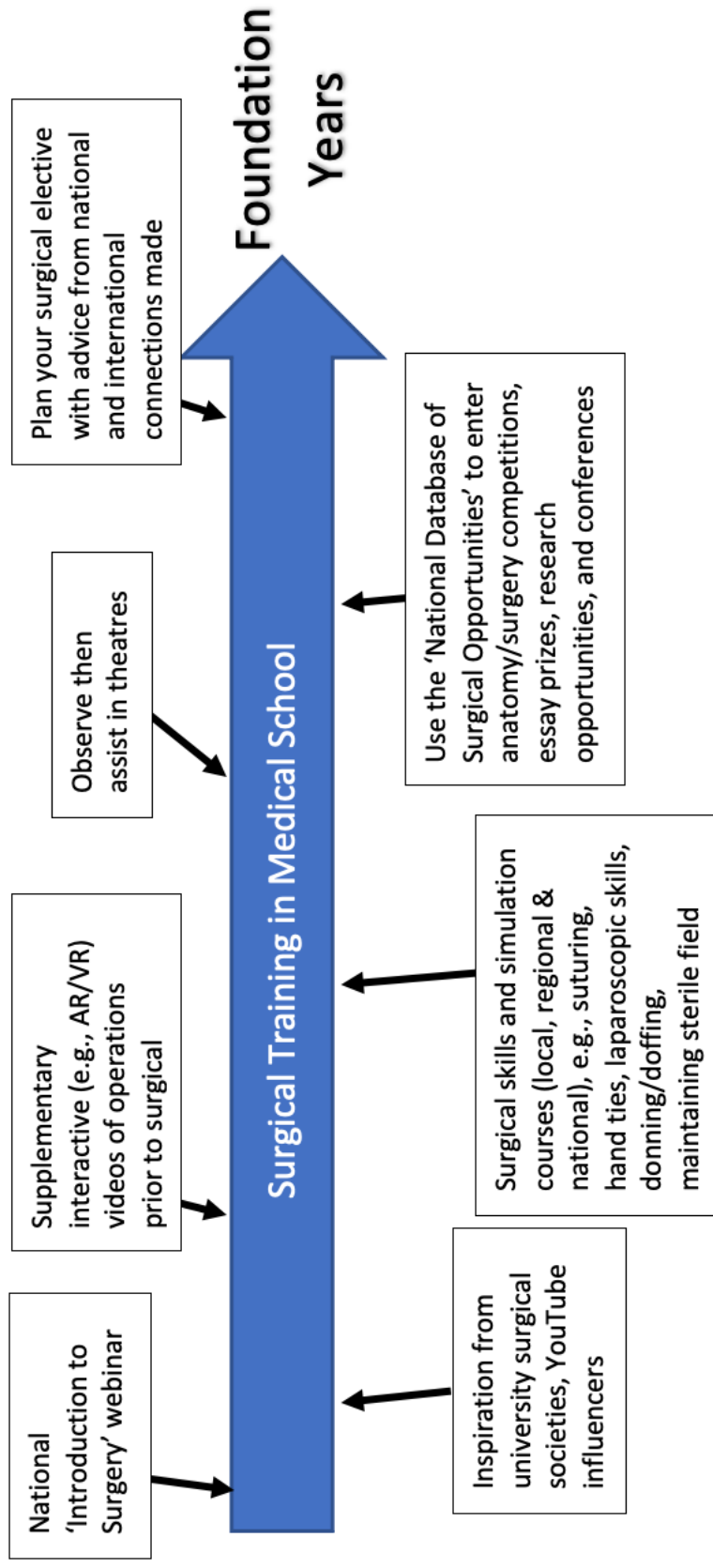

Supplement: Supplementary Figure One — a selection of enhancements placed sequentially along the medical school progression timeline. [file mmc1.pdf]
